# Editorial: Innovative imaging approaches to advance musculoskeletal rehabilitation

**DOI:** 10.3389/fresc.2023.1110409

**Published:** 2023-01-18

**Authors:** Andrew Smith, Adam Kuchnia, Stephan Bodkin, Michael Harris-Love

**Affiliations:** ^1^ Department of Physical Medicine and Rehabilitation, School of Medicine, University of Colorado Anschutz Medical Campus, Aurora, CO, United States; ^2^ Department of Nutritional Sciences, University of Wisconsin-Madison, Madison, WI, United States; ^3^ Department of Physical Therapy and Athletic Training, The University of Utah, Salt Lake City, UT, United States

**Keywords:** imaging, rehabilitation, MRI, ultrasound, DEXA - duel-energy x-ray absorptiometry

Editorial on the Research Topic Innovative imaging approaches to advance musculoskeletal rehabilitation

## Introduction

The utility of imaging biomarkers for medical care continue to rise with advances in technology. These advances hold great potential to progress the field of rehabilitation sciences by identifying novel techniques and methodologies to reduce error, eliminate medical referrals, and accurately predict consequential health outcomes. Within the past two decades, there has been a 131% increase in direct medical costs (treatment, medication, etc.) from musculoskeletal disorders (1). This rising trend is not expected to slow with the projected rise of the aging population (2). The following special topic was composed of emerging scientific research that addresses the following themes: Prediction, Estimation, Automation, and Health Tracking. These themes have been identified to inform and direct the health impact resulting from the rapidly advancing scientific technologies.

### Prediction

For the first published manuscript in this research topic, Bodkin and colleagues aimed to predict lean body mass in obese individuals. Because dual-energy x-ray absorptiometry (DXA) involves ionizing radiation, the authors proposed using available cross-sectional magnetic resonance imaging (MRI) scans to see if they show potential to act as surrogate measures to DXA. They found that cross-sectional MRI measures of muscle were predictive of leg and appendicular lean mass as well as physical function. Thus, this approach may be used as an alternative method to predict outcomes without the need for unnecessary exposure to ionizing radiation.

### Estimation

For the second and third published manuscripts in this research topic, Quinlan and colleagues, as well as Lortie and colleagues (respectfully), investigated innovative imaging approaches to estimate muscle attributes in order to gain clinical insights. The Quinlan manuscript investigated quadriceps muscle volume in patients with end-stage liver disease. The authors found that reducing the number of MRI slices used to manually segment the quadriceps still provides an adequate estimate of muscle volume and was correlated with the skeletal muscle index at the 3rd lumbar vertebrae, a frequently used method for quantifying muscle in the hepatology field. The Lortie manuscript assessed a variety of imaging approaches in healthy adults, older healthy adults, and older adults with non-small cell lung cancer undergoing systemic treatment. They found that MRI-based proton density fat fraction (PDFF) can accurately quantify intra- and extramyocellular percent fat as a reference method for myosteatosis (i.e., muscle quality). The authors also found that ultrasound echointensity may serve as an adequate bedside estimate of myosteatosis measured by PDFF. These studies highlight innovative approaches that can expedite analyses, objectively identify pathology, and translate invasive approaches to the bedside.

### Automation

In the same manuscript described above, Bodkin and colleagues also investigated the ability to train a machine learning model to automate MRI measures. The authors created a convolutional neural network to measure mid-thigh cross sectional area of their lower extremity MRIs, based on manual segmentation. They found strong agreement between the manual measures and the convolutional neural network, suggesting this automated approach may be advantageous in terms of accuracy and time-saving. Bodkin and colleagues have highlighted an approach to expedite analyses and reduce clinician burden.

### Health tracking

Regarding health tracking, the Lortie manuscript, as well as our fourth and fifth published manuscript by Englund and colleagues and Hohl and colleagues (respectively), used innovative imaging techniques to inform tracking of muscle health. Lortie and colleagues found differences in muscle percent fat between young healthy adults and both older healthy adults and adults affected with non-small cell lung cancer. The authors suggest that MRI-based PDFF, and a non-invasive bedside ultrasound approach, may be more sensitive to change over time and more accurately track the progression of muscle wasting in aging and disease, compared to other imaging modalities. Englund and colleagues studied an MRI technique, Intravoxel incoherent motion (IVIM), to measure changes in blood flow in the paraspinal muscles during different types of exercise in healthy adults. The authors found that IVIM may be a useful tool to track muscle blood flow during moderate and high exercise intensities. Hohl and colleagues used MRI-based measures of muscle volume and muscle fat infiltration in the lower extremities to track muscle changes during exoskeleton gait training versus usual care for patients in the acute phase of spinal cord injury. Their findings suggest that early upright mobility, in this case using exoskeletons, may be beneficial to muscle composition and atrophy prevention following injury. These manuscripts undoubtedly have potential to inform rehabilitation programs and implicate myosteatosis in acute and chronic diseases and injuries.

## Summary and future directions

Advancements in biomedical imaging are necessary to manage the emotional, clinical, and financial burden associated with various musculoskeletal conditions in an increasingly older population. As technology continues to improve, there is an opportunity for early detection, diagnosis, and management prior to the development of fulminant and irreversible musculoskeletal damage. This era of biomedical imaging is poised to improve the way clinicians and surgeons make life-changing decisions and will help direct pre- and rehabilitation regimens needed to improve quality of life and healthspan. Innovation related to image acquisition, processing, and analysis is driving widespread recognition and allowing for the ability to expedite time-intensive methods using efficient automated technology, reducing clinician burden and promoting clinical adoption. In order to drive the clinical translational of innovative biomedical imaging, future research should focus on standardization of techniques and analyses between studies and centers in order to harmonize results necessary to drive impactful clinical management. Furthermore, bridging molecular and metabolic aspects of musculoskeletal conditions will provide a mechanistic basis to what is being visualized with these innovative imaging techniques and supports a multidisciplinary approach to improve functional status and morbidity through individualized rehabilitation.

## Author contributions

All authors drafted and reviewed this editorial before submission. All authors contributed to the article and approved the submitted version.
